# Group Coordination Catalyzes Individual and Cultural Intelligence

**DOI:** 10.1162/opmi_a_00155

**Published:** 2024-08-31

**Authors:** Charley M. Wu, Rick Dale, Robert D. Hawkins

**Affiliations:** Human and Machine Cognition Lab, University of Tübingen, Tübingen, Germany; Department of Communication, University of California, Los Angeles, Los Angeles, CA, USA; Department of Psychology, University of Wisconsin–Madison, Madison, WI, USA

**Keywords:** collective intelligence, compositionality, social convention, cooperation, cumulative culture, group coordination, joint action, language, social learning, Theory of Mind

## Abstract

A large program of research has aimed to ground large-scale cultural phenomena in processes taking place within individual minds. For example, investigating whether individual agents equipped with the right social learning strategies can enable cumulative cultural evolution given long enough time horizons. However, this approach often omits the critical *group-level* processes that mediate between individual agents and multi-generational societies. Here, we argue that interacting groups are a necessary and explanatory level of analysis, linking individual and collective intelligence through two characteristic feedback loops. In the first loop, more sophisticated individual-level social learning mechanisms based on Theory of Mind facilitate group-level complementarity, allowing distributed knowledge to be compositionally recombined in groups; these group-level innovations, in turn, ease the cognitive load on individuals. In the second loop, societal-level processes of cumulative culture provide groups with new cognitive technologies, including shared language and conceptual abstractions, which set in motion new group-level processes to further coordinate, recombine, and innovate. Taken together, these cycles establish group-level interaction as a *dual engine* of intelligence, catalyzing both individual cognition and cumulative culture.

## INTRODUCTION

Social learning is a defining feature of human intelligence: we can obtain knowledge from other people that would be costly to acquire on our own (Gweon, 2021). Cumulative culture, meanwhile, is a defining feature of human *societies*: successive generations iteratively build on the innovations of previous generations (Henrich, 2018). A great deal of research has sought to understand the relationship between the two, asking how cumulative culture can emerge from simple social learning strategies (SLS) implemented by individual agents (Boyd & Richerson, 1988; Henrich & McElreath, 2003; Laland, 2004; Tennie et al., 2009). While this line of work has yielded many important insights and resolved puzzling paradoxes, there is still a significant gap between the simplicity of SLS-based transmission mechanisms and the extraordinary scale of the real-world cultural phenomena that remains to be explained.

**Figure 1.**
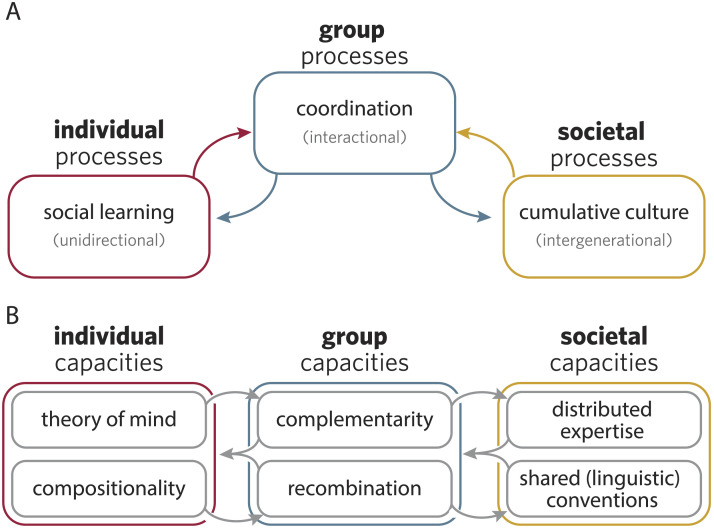
(**A**) We examine processes unfolding at the level of *social learning* mechanisms in individual minds, *coordination* in interacting groups, and *cumulative culture* unfolding across inter-generational societies. (**B**) At each level, we observe bidirectional interactions, creating dual feedback processes.

In the last century, human societies have built cities filled with skyscrapers, organized continent-spanning public education systems, and discovered cures for deadly diseases. Yet, as these same societies grapple with the looming challenges of the next century, such as climate disaster, inequality, and global conflict, it is essential for the cognitive sciences to develop a deeper understanding of how distinctively human collective intelligence emerges (or fails to emerge) from individual minds. In this paper, we argue that explaining the successes and failures of cumulative culture requires a stronger account of the *group-level processes* that mediate between individual agents and inter-generational societies (Figure 1). By the group-level, we mean ad hoc social formations much smaller than societies (e.g., ten people), interacting synchronously over much shorter timescales than those required for cumulative culture (e.g., minutes to hours). The same individual interacts in many different partially-overlapping groups over the span of a day (e.g., with family making breakfast, with coworkers building a product, or with a group of friends just winding down). Importantly, the interface between individuals and societies runs through interacting groups in both directions, giving rise to a characteristic *dual engine* of individual and collective intelligence. Whereas previous work has focused on faithful social transmission as the “ratchet” of cumulative culture (Tennie et al., 2009), here we aim to illuminate the group-level forces pulling at the winch.

In the first half of the paper, we trace one feedback loop linking individual social learning processes with group interaction processes (Figure 1A, left). Specifically, we argue that individual capacities of Theory of Mind (ToM) and compositional representations—absent from SLS-based accounts—facilitate complementarity and recombination in interacting groups (Figure 1B, left). In the reverse direction, the specialized roles and broadened conceptual inventories produced through emergent group capacities shift the computational problem facing individuals, making it easier to track who knows what and engage in targeted social learning. In the second half of the paper, we trace a second feedback loop linking group interaction to cumulative culture at a societal level (Figure 1A, right). In the forward direction, these smaller group-level processes generate stable, institution-like structures necessary for large-scale cultural organization and multi-generational knowledge to persist; in the reverse direction, cumulative culture equips interacting groups with shared knowledge and language, unlocking new capacities for coordination (Figure 1B, right). In closing, we discuss the new insights afforded by this framework and sketch out some research directions it promotes.

## LINKING GROUP COORDINATION WITH INDIVIDUAL SOCIAL LEARNING

A growing body of research has centered around two classes of processes unfolding at the level of individual minds: social learning and the capacity for taking joint action (Brennan et al., 2010; Charbonneau et al., 2024; Chudek et al., 2013; Molleman et al., 2013; Osiurak & Reynaud, 2020; Sebanz et al., 2006; Tomasello et al., 2005; Wang et al., 2018). *Social learning* involves the transmission of information or knowledge between individuals, while *joint action* involves voluntarily cooperating with others in pursuit of a common goal. Human collectives rely on both social learning and joint action for group-level coordination processes. While these processes have been tractable entry points for our models and experiments, we argue that they may not be sufficient to explain how groups reap the full benefits of cumulative culture. A common motif of agent-based models is to show how complex collective phenomena can emerge from extremely minimal assumptions about what is going on inside each individual’s mind (Smaldino, 2017). This approach has yielded remarkable insights, but the simplicity of these individual-level agents may restrict the scope of group-level behaviors that can be explained (Smaldino, 2014).

### Rogers’ Paradox and the Limitations of Oversimplified Agent Models

As an illustrative example, consider an episode from an earlier era, when a phenomenon known as Rogers’ paradox puzzled many researchers. Rogers (1988) reported simulations demonstrating that social learning does *not* necessarily yield benefits over pure individual learning. These simulations presented a type of game theory problem, where agents could either be purely individual learners (with fixed fitness) or pure social imitators (with fitness depending on the number of other imitators in the group). Individual learning is assumed to yield a constant payoff rate, but the payoffs for the imitation strategy depend on the number of other imitators in the population. That is, imitation pays off when there are few imitators, but fails dramatically when everyone is imitating other imitators, due to maladaptive information cascades (Bikhchandani et al., 1992; Toyokawa et al., 2019; Tump et al., 2020). Noisy individual responses get amplified by imitation and start to swamp the signal, as when a single jumpy wildebeest causes the whole herd to spontaneously stampede. Rogers found that mixed ratios of individual learners and imitators were evolutionarily stable, but surprisingly, these groups performed no better than a population of entirely individual learners.

In reaction to Rogers’ paradox, a slew of research suggested modifications to the simulations, showing that structured reward environments (Kobayashi & Ohtsuki, 2014) and more sophisticated social learning strategies (Boyd & Richerson, 1995; Enquist et al., 2007; Kameda & Nakanishi, 2002) can make the paradox disappear, with social learning yielding additive benefits over individual learning after all. Key cognitive mechanisms that support cumulative social learning include adaptive switching between strategies (Boyd & Richerson, 1995; Enquist et al., 2007; Kameda & Nakanishi, 2002) and selective imitation (Garg et al., 2022; Hawkins, Berdahl, et al., 2023; Wu et al., 2023), which minimize maladaptive copying and information cascades. Of course, Rogers’ results were unintuitive enough to be considered a paradox, spurring further developments aimed at resolving it. Unfortunately, we don’t always have such clear intuitions for more complex behaviors, and our findings may *not* strike us as a paradox in the same way. In this sense, Rogers’ paradox may be taken as a case study for how enriching the set of individual mechanisms under consideration can vastly expand our explanatory power at the population-level. Conversely, oversimplifying those mechanisms can place significant limits on the explanatory range of the model.

### Section Summary

In the rest of this section, we map out two commonly overlooked group-level phenomena: (i) the ability of groups to adaptively take on complementary and specialized roles, and (ii) the ability of groups to collectively search and propagate novel solutions by recombining socially acquired information with private knowledge. Both of these facilities depend upon more sophisticated mechanisms of individual cognition than typically captured in SLS-based models. Specifically, they depend upon (1) *Theory of Mind* (ToM) capacities to make inferences about the hidden mental states of others, and (2) *compositional representations* to recombine knowledge structures. The bidirectional interaction between individual social learning mechanisms and group interaction is the first of the dual engines driving the individual and collective intelligence.

### Theory of Mind Facilitates Complementarity in Interacting Groups

#### Complementarity in Interacting Groups.

Complementarity refers to the ability of an interacting group to flexibly adopt *specialized roles* while working toward a joint goal (Dale et al., 2011; Goldstone et al., 2024). While our focus will be on interacting groups at the scale of a basketball team or operating theater, complementarity pervades nearly all levels of human society (Durkheim, 1893), from hunter-gatherer communities (Hooper et al., 2015; Kelly, 2013), to diverse organizations of working artisans and craftspeople in the 16th–18th centuries (Rappaport, 2002; Thompson, 1964), to the highly stratified market economies we live in today (for some discussion: Cazzolla Gatti et al., 2020; Falandays et al., 2022; Sterelny, 2007; Sutton, 2013). To be clear, complementarity is not necessarily beneficial for all individuals involved, or even for the group as a whole. Indeed, it is often the basis for oppressive inequality and social stratification (Henrich & Boyd, 2008; O’Connor, 2019). Whether for good or ill, it is clear is that the ability to infer and adapt to different roles in different groups is a core feature of human sociality, which must be understood to navigate the challenges faced by modern societies (Smaldino, 2014). As we will argue when we consider larger *societies* below, the ability of small ad hoc groups to adaptively organize into complementary roles on the fly is key to enabling larger-scale cultural transmission and distributed expertise at the societal level.

Foundational research on group coordination has largely focused on reciprocity of prosocial behaviors (Henrich et al., 2001; Henrich & Muthukrishna, 2021) rather than complementarity, relying heavily on game theory dilemmas where individuals need to match their actions for maximum benefit. For instance, in the Prisoner’s dilemma (Flood et al., 1950), two individuals stand to gain more if both are committed to staying silent rather than both betraying each other. But without guarantees about the other’s choice of action, people are often motivated to betray the other, leading to lower payoffs when both betray. In such settings, cooperation is often shown to emerge through evolutionary mechanisms such as kin selection (Hamilton, 1964), impure altruism (Andreoni, 1989), third-party punishment (Fehr & Fischbacher, 2004), or even pseudo-reciprocity (Bouhlel et al., 2018; Brown et al., 1991) which all describe an incentive structure for undertaking prosocial rather than narrowly self-interested behaviors.

It is less clear how these mechanisms explain the way that groups self-organize into complementary roles over shorter (non-evolutionary) time scales. Reciprocity requires actions to match, while complementarity actually often requires actions to differ in coordinated ways (Fiske, 2000). Distinct profiles of beliefs and knowledge must be distributed throughout the population. Anthropological studies indicate that even groups formed within small-scale egalitarian societies tend to differentiate a set of leadership roles to facilitate coordination (Glowacki & von Rueden, 2015; Johnson & Earle, 2000; Read, 1959). For example, a classic study by Elinor Ochs (1988) observed that mothers in a Samoan village divvy up different caregiving tasks among younger caregivers (see Scheidecker, 2023). How, then, does this kind of adaptive complementarity arise from individual cognition? Is it simply an evolutionary consequence of isolated groups of social learners copying one another (Henrich & Boyd, 2008), or are there deeper cognitive principles at play?

#### Theory of Mind in Individuals.

The majority of research on social learning strategies has focused on simple mechanisms for imitation (Heyes, 2002; Laland, 2004; Legare & Nielsen, 2015; Tomasello, 2000; Whiten & Ham, 1992), with only slightly more sophistication than Rogers’ imitators. However, humans are capable of much richer, more flexible inferences about others’ hidden mental states (Frith & Frith, 2012; Gweon, 2021). The capacity to make these inferences is commonly referred to as Theory of Mind (ToM), and there is abundant evidence that humans use ToM to make educated guesses about the values, goals, and beliefs that others hold about the causal structure of their environment (Baker et al., 2017; Jara-Ettinger et al., 2015). Of course, having access to this capacity does not mean we necessarily rely on it in all contexts (Charpentier et al., 2020; Hawkins et al., 2021) nor expect it to be deployed in the same way across all cultures (Barrett et al., 2016; Barrett & Saxe, 2021; Curtin et al., 2020). Indeed, we may judiciously trade off more expensive inferential social reasoning with cheaper “snap judgements” (Wu et al., 2022).

Yet, having such meta-cognitive capacities at our disposal makes social information much more useful than any imitation-based strategy could. For example, individuals are able to account for shared knowledge (Brennan et al., 2010; Fränken et al., 2022; Whalen et al., 2018), modulate generalization based on whether demonstrations were accidental or pedagogical (Csibra & Gergely, 2009; Gweon et al., 2010), and distinguish context-specific information from more generalizable information, effectively learning from people with different goals (Witt et al., 2024) and perhaps even glean useful information from failed or imperfect solutions (Wu et al., 2022).

#### Theory of Mind Facilitates Complementarity Through Role Inference.

Here, we hypothesize that Theory of Mind facilitates qualitatively greater group complementarity than standard SLS (Laland, 2004) or assortment mechanisms (Apicella & Silk, 2019) would predict. While these simpler individual mechanisms are able to account for a range of collective phenemomena (Kendal et al., 2018; Laland, 2017), they lack key features present in human coordination, such as selective imitation via inference mechanisms (Hawkins, Berdahl, et al., 2023) and adaptive role delegation (Wu et al., 2021). And while even social insects occupy specialized roles (Beshers & Fewell, 2001) and can transmit culture (Bridges et al., 2024), human coordination is distinguished by how flexibly we can adopt new, ad hoc roles as required by the task at hand (Dale et al., 2011; McCarthy et al., 2021; Wu et al., 2023), allowing us to work together in novel ways that insects cannot: building skyscrapers or continent-spanning initiatives, and traveling to the moon (Laland, 2017).

Despite being associated with the kind of strategic one-shot reasoning studied in game theory (Meijering et al., 2012; Yoshida et al., 2008), Theory of Mind also provides a critical foundation for more sophisticated longitudinal cooperation via joint reasoning about *roles*. Even simple imitation-based models can display specialization to some degree (Dale et al., 2013; Wu et al., 2023), with the push (Setzler & Goldstone, 2020) and pull (Frey & Goldstone, 2018; Goldstone & Ashpole, 2004) of social information providing low-level self-organizing mechanisms for specialization (Goldstone et al., 2024). Yet a key feature of successful joint-action coordination is to be able to anticipate the actions or intentions of others on the fly (McEllin et al., 2018; Richardson et al., 2007; Sebanz et al., 2006). This kind of ad hoc role assignment (Genter et al., 2011) depends on the ability to consistently track other individuals’ distinct goals, skills, preferences, and beliefs. This is precisely the advantage of ToM mechanisms (Jara-Ettinger et al., 2015; Wu et al., 2022), which enable a more effective form of coordination.

**Figure 2.**
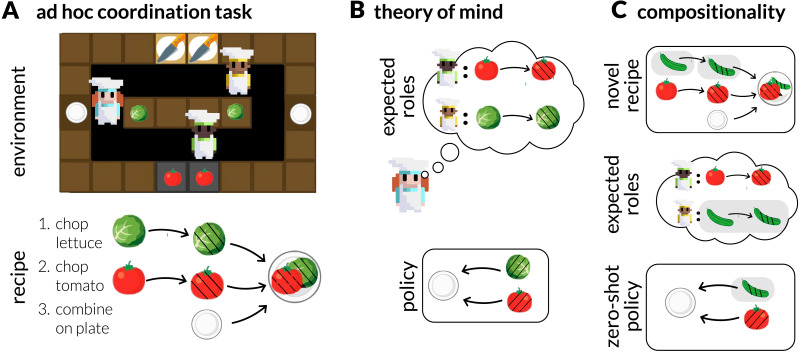
(**A**) Groups coordinate to solve challenging ad hoc coordination problems by (**B**) decomposing the group into distinct roles using Theory of Mind, and (**C**) decomposing the task into subgoals. Graphical elements from Strouse et al. (2021) and Wu et al. (2021).

A strong demonstration was recently provided by Wu et al. (2021), who studied interacting groups of agents in a collaborative cooking task based on the game *Overcooked* (Figure 2A). The problem of group coordination (e.g., successfully making a salad) can be formalized in a Bayesian framework, where social inference based on Theory of Mind was found to be crucial for allowing groups to distribute specialized roles and collaboratively solve tasks with many interlocking parts and dependencies (see also Carroll et al., 2019; Davis et al., 2021; Kleiman-Weiner et al., 2016; Tang et al., 2022). There is also evidence that these types of interactions may spontaneously engender “interpersonal synergy”, in which participants do not simply synchronize, but build routines that can be distinct and complementary (Fusaroli et al., 2012; Fusaroli & Tylén, 2016). In sum, ToM allows groups of interacting agents to self-organize into complementary roles that facilitate more effective coordination than simpler social learning strategies.

### Compositional Representations Facilitate Group-Level Recombination

#### Recombination Drives Innovation in Interacting Groups.

Successful group problem-solving is often characterized by the recombination of existing solutions (Creanza et al., 2017b; Henrich, 2009; Majchrzak et al., 2012; Rogers, 2010; Youn et al., 2015), leading to “bursty” trajectories that proceed in fits and starts (Kolodny et al., 2015; Miu et al., 2018). Recombination allows groups to explore a larger solution space by drawing upon successful components of existing solutions; agents are able to innovate without losing their progress or backing themselves into a corner. For example, in one study, Derex and Boyd (2016) tasked groups with discovering the most effective combination of ingredients for a virtual remedy. Despite the complex fitness landscape, small partially-connected groups were able to sustain a diversity of solutions and discover effective new combinations more effectively than fully-connected groups (see also Wisdom et al., 2013). These findings are also supported by recent computational models, where it is observed that agents implementing a *partial-copying* policy and randomly borrowing elements of others’ solutions are better able to avoid premature convergence (Barkoczi et al., 2016; Campbell et al., 2022).

Importantly, when others’ solutions are inferred via social learning, these inferences do not need to be exact for their outcomes to be usefully recombined. Even imperfect or incorrect inferences about causal structure can help generate new breakthroughs at the group level (Wu et al., 2022). For example, the “nixtamalization” of corn flour (a complex process involving adding a caustic agent to corn kernels, which was only recently discovered to unlock greater bio-availability of nutrients) is often touted as evidence for the power of trial-and-error combined with selective cultural preservation (Henrich, 2018). However, inferring the wrong causal structure about this process may nevertheless allow for a greater rate of innovation in groups than random mutations would predict. For instance, (incorrectly) reasoning that the purpose of the caustic agent is to ritualistically remove “impurities” may suggest soaking the kernels for longer or rinsing more thoroughly when finished, which may improve the process. Even though the inferred causal structure is technically incorrect, building a causal representation of the problem through ToM (i.e., by rationalizing the underlying motivations of another actor; Cushman, 2020) or considering others’ “reasons for action” (Barrett et al., 2016) may allow for greater strategic exploration (Vélez et al., 2022).

#### Individuals Represent Complex Knowledge Via Compositional Structures.

We now turn to another important aspect of individual cognition that is underrepresented in agent-based models of social learning: compositional representations that, we argue, allow groups to exchange and recombine diverse knowledge more effectively than existing SLS-based models predict. Compositionality has long been considered a singular feature of human cognition (Dehaene et al., 2022; Frege, 2010), and can be defined as the capacity to represent complex problems based on the meaning of their parts and how they are combined. Thus, compositionality is deeply related to the notion of a “Language of Thought” (Fodor, 1983; Fodor & Pylyshyn, 1988; Piantadosi et al., 2016), and has primarily been studied in the context of asocial tasks, including learning (Feldman, 2000; Simon, 1972), memory (Planton et al., 2021), and navigation (Rubino et al., 2023). Most importantly for interacting groups, there is extensive evidence that individuals decompose complex tasks into sets of “subgoals” for more effective planning (Correa et al., 2023; Huys et al., 2015). For instance, the individual goal of making a latte can be broken down into relevant subgoals, such as grinding beans, boiling water, and frothing the milk (Botvinick & Weinstein, 2014; Jackendoff, 2007).

Representing this task in terms of compositional subgoals allows us to selectively intervene at sub-branches when we run into an issue (e.g., if the beans are in an unexpected cupboard, we don’t need to reboil the water), as well as to more effectively recombine techniques at different sub-branches to generate innovations (e.g., we can experiment with a new grind setting while keeping the rest of the process fixed; Muthukrishna & Henrich, 2016). While there has been a recent resurgence of interest in understanding compositionality in asocial contexts (Amalric & Dehaene, 2019; Rubino et al., 2023; Sablé-Meyer et al., 2021; Schwartenbeck et al., 2023), here we focus on the social and cultural consequences of compositional representations. Just as individual representations of the world can be compositional in nature (i.e., decomposible into primitives and productively recombined; Kurth-Nelson et al., 2023; Schwartenbeck et al., 2023), so too might beliefs inferred from (or about) others (Uchiyama et al., 2023).

#### Compositional Representations Facilitate More Effective Recombination.

We hypothesize that the leaps and jumps we observe in cumulative culture (Derex & Boyd, 2016; Kolodny et al., 2015; Miu et al., 2018) are better explained by individual agents with compositional representations rather than simply assuming *random* mutations in copying, as many classical models do (Creanza et al., 2017a; Henrich & McElreath, 2003; Richerson & Boyd, 2008). That is, compositionality should help agents more flexibly integrate socially acquired (public) information with their own structured (private) understanding of the problem, rather than only adopting noisy versions of existing solutions. When knowledge is represented in an appropriate compositional structure, hybrid solutions can be obtained by swapping structurally coherent fragments of individually acquired knowledge structures out with socially inferred fragments (Cano et al., 2023; Jern & Kemp, 2013; Muthukrishna & Henrich, 2016; Uchiyama et al., 2023), achieving leaps of innovation that are greater than the sum of their parts. For example, a Japanese chef might acquire an understanding of which ingredients pair well with avocado from observing Mexican or Californian cuisine, and plug this fragment in while retaining the broader structure of their sushi training to generate new culinary innovations like the California roll (introduced by Hidekazu Tojo in 1970s Vancouver). It is difficult to see how such innovations could emerge from the usual incremental tweaks predicted by models of blind trial-and-error copying (Acerbi et al., 2022; Legare & Nielsen, 2015).

### Completing the First Feedback Loop

#### Co-Evolution of Complementarity and Recombination.

We have highlighted how two (relatively sophisticated) features of individual cognition facilitate coordination in interacting groups. Specifically, we hypothesized that ToM facilitates complementarity in group roles while compositionality facilitates recombination in group search. In this section, we argue that these pathways actually form a *feedback loop*, unlocking shifts in individual cognition as well. We start by observing that complementarity and recombination are catalysts for one another *within* the group level. On one hand, the distribution of more diverse knowledge through complementarity can, in turn, increase the pool of abstract structures that can be drawn upon for recombination (Fjaellingsdal et al., 2021). On the other hand, recombination yields a constantly expanding space of concepts and goals for individuals to potentially specialize in, hence affording greater complementarity of specializations. In this way, although ToM and compositionality are distinct cognitive capacities, they may work together (along with simpler forms of social transmission and individual learning) to maintain diversity and flexibility within groups.

#### Emergent Group Capacities Shift the Computational Problem Faced by Individuals.

What consequences, then, do complementarity and recombination have at the individual level? How is this a feedback loop, as opposed to a bottom-up process? We suggest three ways that these group-level capacities might change the fitness landscape for individual intelligence by introducing new computational constraints (or weakening existing constraints).

First, to the extent that the group develops a wide variety of complementary roles (e.g., butcher, baker, candlestick maker), each individual no longer needs to maintain the entirety of their society’s knowledge in order to survive, thus easing cognitive load and allowing the agent to pursue deeper expertise in specialized domains (Genter et al., 2011; Pradhan et al., 2012). Future empirical work can test these predictions by manipulating the costs of deploying ToM (e.g., cognitive load) or the degree of compositionality in a task, to see whether individual specialization will be reduced. Second, to the extent that individuals in a group have tacitly agreed on the same representation of complementary roles (i.e., the same decomposition of their task), they may use ToM to track who has expertise in which areas, and thus engage in “on-demand” or “asynchronous” processing to retrieve needed fragments only when relevant (Hollingshead, 2000). An empirical test of this prediction could be provided by manipulating the stability of interaction partners, with the expectation that less predictable partner pairings will lead to an impaired ability to exchange social information. Third, group recombination endows each individual agent with a combinatorially expanded conceptual repertoire (i.e., through combining fragments of other socially observed solutions), facilitating new ways of approaching the problems they individually encounter (Pradhan et al., 2012). This hypothesis predicts that agents “trained” in interacting group contexts should generalize more effectively to downstream individual problem-solving tasks, with the magnitude of the effect dependent on the degree of specialization and recombination in the training group. Thus, when the distinct functional pressures at the individual and group levels are considered together, we begin to see their co-evolution as one important engine of social intelligence.

## LINKING GROUP COORDINATION WITH CUMULATIVE CULTURE

In the previous section, we sketched out an account of the feedback loop between individual and group-level processes. This loop traverses through two core capacities of individual cognition that are not typically captured by simple imitation-based models of collective behavior: ToM and compositionality. We then showed how the interplay of these individual-level capacities may enable the emergence of new group-level capacities. First, by using ToM to flexibly infer the intentions and anticipate the actions of other group members, agents are able to plan their part in joint actions, thus executing complementary roles. Second, the compositionality of individual representations allows groups to quickly recombine abstract pieces of knowledge, splicing structured fragments of their own knowledge together with those inferred from social partners. When these group-level capacities begin to catalyze one another, we saw that they also shifted the computational problem faced by individual agents: agents can begin to tacitly depend on social expectations about specialized roles and build on a larger repertoire of concepts.

### Section Summary

**Figure 3.**
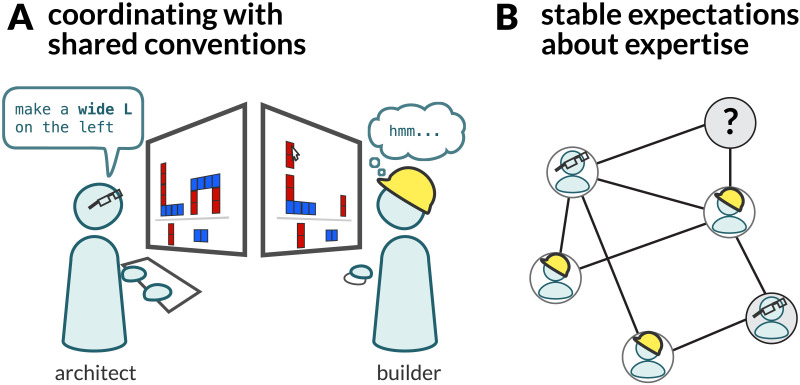
(**A**) The ability to send explicit communicative signals with culturally acquired meanings helps to coordinate expectations in groups. (**B**) Culturally transmitted knowledge about the distribution of expertise in a population helps to seed priors for group interaction. Adapted from McCarthy et al. (2021).

In this section, we now extend our analysis to consider a second feedback loop between local group-level processes and the larger-scale cultural processes that are characteristic of human societies (Henrich, 2018; Laland, 2017; Tomasello, 2000). Rather than analyzing the impact of cumulative culture directly on *individuals* (e.g., inductive biases shaping learning; Kalish et al., 2007), we suggest that the level of *interacting groups* may provide a more natural level of analysis (Hawkins, Franke, et al., 2023). The functional need to rapidly align conceptual representations and role specializations within small groups places strong pressure on the development of collective solutions like shared (linguistic) conventions and structured distributions of expertise throughout the population (e.g., Croft, 2000; Figure 3A). These emergent products, in turn, become cultural technologies that allow agents to better navigate new group compositions, without needing to re-negotiate roles anew in each generation. In particular, the capacity to communicate explicitly in a shared language about relevant concepts and roles allows groups to interact more effectively (Figure 3B). We propose that this second feedback loop, where cultural technologies allow for cumulative improvements in the way ad hoc groups are able to coordinate, plays an important role in the collective intelligence of our species. As above we will begin with the consequences of these cultural capacities on *group coordination*, and finally complete the feedback loop by examining how the computational challenges arising at the group level place functional pressures (and affordances) on cultural transmission.

### Cultural Conventions Facilitate Complementarity in Groups

#### Shared Language Serves as a Prior for Coordinating Joint Action.

One of the most powerful culturally-transmitted tools for group organization is a set of *shared conventions* allowing agents to explicitly communicate using language. Communication is a form of joint action that allows groups to establish joint commitments and plan toward joint goals (Clark, 1996, 2006). When endowed with a set of culturally-transmitted conventions for the meanings of words and phrases, groups are able to coordinate their expectations and actions, even when their interactions are brief and relevant concepts are unfamiliar (Bangerter & Clark, 2003; Clark, 2005; Clark & Wilkes-Gibbs, 1986; Hawkins, Frank, & Goodman, 2020). For example, McCarthy et al. (2021) examined the convergence of new conceptual and linguistic representations across just twelve trials in a tower-building task (Figure 3A). One participant, the *architect*, was privately shown a blueprint of a tower, which the other player, the *builder*, needed to construct. Architects gradually shifted from giving primitive block-level instructions like “place a red block on top of the blue block” to more abstract instructions like “make a skinny L” or “build an arch,” which were grounded in novel procedural chunks. Like these participants, members of all kinds of groups engage in communication as an extensive co-creative activity. New conventions are not just throwaway mappings between a word and target concept; they become first-class primitives that can be compared with other meanings and systematically transfer to nearby targets (Eliav et al., 2023). People harness existing conventions to align on new concepts and new conventions for talking about them, which then serve as the building blocks for new, more complex tasks down the road (Effenberger et al., 2021), thus producing path-dependent evolutionary trajectories of cumulatively refined conventions (Buskell et al., 2019).

#### Shared Language Endows Groups With Shared Conceptual Primitives.

In addition to its use in joint action, sharing a language also endows groups with a common set of concepts and abstractions to draw on. Heyes (2018) likens language to a “cultural gadget” facilitating complex reasoning, which emerged and evolved through cultural forces. Much as a mangrove tree’s roots grow and accumulate “forest islands” around the tree, language expands from a base of conceptual material to grow a forest of culturally-transmitted abstractions (A. Clark, 1998). But if language is a culturally-transmitted tool, what kind of tool is it? We may productively think of a linguistic utterance as more akin to a computer program than an axe or a hammer (Cano et al., 2023; Wong et al., 2023). Axes and hammers are constructed to solve specific problems (e.g., chopping down trees or hitting nails) in the same way that a particular computer program is constructed to solve specific problems (e.g., calculating a tip percentage or moving a robot’s limbs). But programs have the added benefit of being *compositional recipes* for behavior, drawing from larger, more expressive libraries of abstractions (e.g., functions, procedures, definitions). It is in this sense that Lupyan and Bergen (2016) argue that language is a means to “mutually program” one another to act in the world (see Sumers et al., 2024, for a recent formalization of this process). Languages encode composable, embodied representations. When shared between agents, these representations systematically guide others’ engagement with the world (cf. Lupyan & Clark, 2015), allowing behavior to become more tightly time-locked and tuned to the context (Dale et al., 2011).

#### Shared Language Directly Encodes Beliefs About Social Roles.

Further enhancing group complementarity and the assignment of roles, language can explicitly encode social roles, with discussion about who is doing what (Abney et al., 2021; Fusaroli et al., 2012; Paxton et al., 2022), about social network structure (Barkoczi et al., 2016; Dubova et al., 2020; Sloman et al., 2021), and about institutions of leadership (Pietraszewski, 2020; Shaw & Hill, 2014; Sumpter, 2009). Some languages use different pronouns to encode relative social status, closeness or formality, as in French with the more formal second-person pronoun *vous* used for those perceived as having higher social status, while *tu* marks a kind of closeness or intimacy (Agha, 1994; L’Huillier, 1999). These features are sometimes thought to be politeness conventions made mandatory in grammatical structure—different pronouns require different verb conjugations—which force groups to confront the functional problem of recognizing and coordinating beliefs about the social status of members. Many languages also directly encode evidentiality (de Haan, 2013) using a grammatical affix on the verb that expresses whether an event was directly perceived by the communicator (“I saw that”) or obtained second hand (“I was told that”). These grammatical encodings reveal another way individual ToM meets with culturally-transmitted language representations: they directly expose an individual’s inner social beliefs for all to see or hear.

### Distributed Societal Expertise Facilitates Recombination in Groups

#### Global Networks of Expertise Are Distinct From Local Division of Labor.

A second emergent artifact of culture is the highly distributed network of expertise built up across society (Figure 3B). As the body of culturally-transmitted knowledge grows, individuals repeatedly engage in distinct domains of action over long periods of time. Locally interacting groups may then leverage shared representations of these relatively stable niches (“who knows what”: Heyes, 2016) as part of meta-cognitive strategies to plan and act together more effectively. Explicitly defined roles and institutions make it easier to access specialized knowledge on demand. At one level, these societal networks of expertise may simply appear to be an outgrowth of the local divisions of labor discussed earlier in small, interacting groups. However, the vast gap in spatial and temporal scales between the local group and global society entails qualitatively different phenomena, and the precise relationship between them requires an explanation. Societies are too large for every individual to directly interact with everyone, requiring an inductive leap that extends expectations to complete strangers (Hawkins, Franke, et al., 2023). We hypothesize that the broader distribution of expertise that emerges at the societal level is a cultural technology that evolves to serve the functional needs of transmission and collaboration in small groups, enabling more synergistic group-level interactions. Under this account, the structured distribution of expertise generated by societal processes predicts greater rates of innovation than in settings where each individual needs to independently seek out interaction partners without the benefit of institutional structures.

#### Distributed Expertise Supplies Diverse Building Blocks for Group Recombination.

First, distributed networks of expertise turn local groups into *laboratories of conceptual innovation* where diverse perspectives interactively experiment with candidate policies, leading to more powerful recombination (Campbell et al., 2022; Pradhan et al., 2012; Wisdom et al., 2013). Critically, when paired with the combinatorial power of a shared language, interacting groups can collectively simulate solutions through discussion and debate without requiring immediate behavioral commitment (Bickerton, 1990). In other words, expertise can be remixed and recombined through explicit verbal communication rather than through real-world trial-and-error. The best elements of different policies or perspectives can be tentatively combined in order to test whether a stronger composite solution can be produced. This solution then becomes part of the conceptual repertoire each individual carries into other small groups in the future, planting the seeds for greater global diversity. It is not always clear, however, how much conceptual variability is good for a group: agent-based simulations have revealed an apparent “paradox of diversity” (Schimmelpfennig et al., 2022; Sulik et al., 2022), where the ideal balance of building blocks depends on the group’s network structure (Barkoczi et al., 2016) and the forms of social learning they are using (Barkoczi & Galesic, 2016). Yet, much of this work assumes purely imitation-based social transmission. The more complementary and compositional processes we describe may afford even greater benefits for diversity.

### Completing the Second Feedback Loop

#### Meta-Learning Across Group Interactions.

A key insight from recent computational work is that society-level roles and conventions may be formally understood as *meta-learned* solutions distilling many distinct episodes of local group interaction (Hawkins, Franke, et al., 2023). As described above ad hoc roles and conventions emerge within each locally interacting group through ToM. However, these ad hoc roles and conventions are ephemeral, only lasting as long as the interaction itself. The framework of meta-learning (whether implemented in a hierarchical Bayesian model, or a neural network; Hawkins, Kwon, et al., 2020; White et al., 2022) helps explain how the functional demands of group coordination in local episodes can shape global culture over longer timescales. Meta-learning thus calibrates each agent’s linguistic and social priors to the distribution of coordination problems that commonly arise when navigating a variable, non-stationary landscape of potential interaction partners. Whereas short-term plasticity is required for agents to rapidly adapt background expectations to their current group of partners, long-term stability is required to abstract away policies that tend to work well on average across many groups.

The interplay of these short and long timescales provides a driving force for the evolution of cultural capacities like language (Brochhagen et al., 2023). The meanings encoded in linguistic conventions have been meta-learned to travel well across diverse contexts and populations. In this way, cultural transmission through repeated group interaction begets new cultural technologies that make future group interaction more efficient. A language’s lexicon expresses thousands of conceptual distinctions, from feelings to foods. An active area of investigation in the study of language evolution concerns the relationship between the size and conceptual structure of a community’s lexicon and aspects of their cultural context (Reali et al., 2018; Regier et al., 2016; Tria et al., 2012). For example, the argument structure for verb constructions involving “give”, “take”, “borrow,” or “promise” encode high-level relational templates for common types of interactions between agents (Goldberg, 2019). Thus, meta-learning extracts the generalities of group-level interactions and encodes them as cultural tools for facilitating coordination and cooperation.

#### Local Distributions of Knowledge Are Amortized Through “Desire Paths.”

Likewise, the structure of human knowledge networks are another form of cultural technology that catalyze group-level interactions. Distributed knowledge networks, such as the web of scientific exchange or global supply chains, connect hubs of specialized knowledge with one another in a complex logic of interactions. These knowledge networks are largely developed in a collaborative and self-refining manner, with the connections encoding amortized computations for facilitating efficient exchange between hubs. For instance, “desire paths” (Goldstone et al., 2006; Goldstone & Roberts, 2006) provide a good metaphor for how previously traversed routes between specialized nodes create self-reinforcing connections. Just as the strip of trampled grass across a campus lawn amortizes previous solutions (for finding a faster route to class), each new knowledge seeker does not need to solve the complex search problem of finding the best expert from scratch (Dasgupta & Gershman, 2021; Gershman & Goodman, 2014). Previous solutions are amortized in the institutional and cultural memory of communities, from legal precedents to university programs to corporate protocols. Yet previous connections can still be adaptively bypassed if a better solution is found, dynamically adjusting the structure of our knowledge networks to better link up specialized hubs and tuning the diversity for the problem at hand. In sum, cumulative culture creates specialized knowledge hubs together with flexible transmission structures designed to efficiently connect individuals with the knowledge they seek.

## CONCLUSION

We have argued that understanding the relationship between social learning (at the level of individual minds) and cumulative culture (at the level of societies) requires an account at level of the interactive group-level processes that mediate between them. Crucially, this mediation runs both ways, leading us to identify a pair of feedback loops. On one hand, individual-level capacities including compositionality and theory of mind (ToM) reasoning facilitate group-level coordination through complementarity and recombination. On the other hand, societal-level products of cumulative culture provide us with new tools, such as language and distributed knowledge networks, which unlock new methods to further coordinate, recombine, and innovate. While it has always been tempting to explain cultural evolution through as a massive scaling of individual cognitive processes, group-level coordination ought to be an important theoretical stepping stone in this endeavor. We close by sketching out several future directions for research suggested by our framework.

### The Emergence of Power and Structural Inequality.

We have described an engine that is remarkably successful at accelerating social intelligence through cumulative culture, and our examples drew from fairly innocuous domains like collective search or cooking. But engines are blind to where they’re going. We have already observed that the dynamics driving beneficial complementarity also contain the seeds of systemic inequality (O’Connor, 2019). However, many existing models lack an adequate notion of (legitimate or illegitimate) power (Saxe, 2022): as agents differentiate, some may capture an outsized role in the group’s decision-making, even exerting social control over the roles others play in the group. That is, the same complementarity that facilitates group-level coordination when incentives are aligned (e.g., depending on someone else to grow food so we can do other things), also allow certain individuals or organizations of individuals to slip in and manipulate the structure of the group, reinforcing their power. In this way, the cultural engine may be turned toward solving problems that are counter to the democratic interests of the collective, or ignore our most pressing existential problems altogether (i.e., climate change). We hope our framework, centering interactive group processes, provides a pathway for more rigorously investigating power and status dynamics in future research.

### The Emergence of New Group Identities and Institutions.

One of the most dramatic and underexplored consequences of distributed expertise at the societal level is the rearrangement of social connections. As distinct, coherent clusters of expertise take shape in the overall population, domain experts develop communal lexicons (H. H. Clark, 1998) and begin to be perceived by others (and themselves) as a unified social group (Gershman & Cikara, 2020; Hacking, 1996; Sparti, 2001). Areas of specialization may develop unique cultural institutions (e.g., graduate programs, companies, unions) that take responsibility for transmitting the required knowledge to new would-be-specialists (Gallagher & Crisafi, 2009), which tightens in-group connection and differentiates them from other out-groups. For example, no one person in the world fully understands how every part of a modern computer works. It takes experts on microchips and transistors (“electrical engineers”) working in concert with systems engineers, software engineers, user interface designers, and so on, to piece together the now-commonplace computer. Teams are often (self-)organized with explicit mutual knowledge of who belongs to which respective groups; when a particular problem arises, everyone knows which complementary specialist to talk to about it (Maglio et al., 2009). Being able to rely on others’ cooperation in this way allows even greater specialization, and more elaborate team compositions, which we hope future modeling efforts will tackle. We also hope our framework will help make better predictions about which groups will be more synergistic with one another, supporting social “mergers” or coalitions when acting together.

### The Emergence of Long-Run Contradictions From Social Feedback Loops.

While the full sweep of economic and social phenomena emerging from the cognitive mechanisms we describe are clearly beyond the scope of this article, our framework is largely in line with the long tradition of social theorists grappling with the internal contradictions produced by engines of culture. Often, agent-based models focus on explaining the immediate, positive outcomes of cultural evolution but do not grapple with evidence of negative outcomes that often follow in practice. For example, while models of cumulative culture predict that our tools for communication should only increase mutual understanding and better approximations of the truth, in practice, we observe increasing polarization of beliefs and susceptibility to misinformation (Brady et al., 2023). While group complementarity should lead to rising intelligence and cognitive capacity for all individuals, in practice, it has historically led to mass *de*skilling (Braverman, 1998) and exploitation through wage labor. While distributed expertise should lead to increased accessibility of the cumulative knowledge of a society, in practice, it has facilitated gate-keeping and inequality of access to information. Indeed, while we have focused on the functional engines of group *cooperation*, social interaction is also frequently characterized by *conflict*, which exerts its own functional pressures (De Dreu & Gross, 2019; Paxton & Dale, 2013). We hope that circling back to some of these contradictions from the perspective of modern cognitive science will provide new analytical tools to illuminate and intervene upon the societal challenges that human groups continue to face.

## ACKNOWLEDGMENTS

We are grateful for helpful comments from Claudio Tennie, Ryutaro Uchiyama, Wataru Toyokawa, and Clark Barrett on an early draft of the manuscript.

## FUNDING INFORMATION

CMW is supported by the German Federal Ministry of Education and Research (BMBF): Tübingen AI Center, FKZ: 01IS18039A and funded by the Deutsche Forschungsgemeinschaft (DFG, German Research Foundation) under Germany’s Excellence Strategy–EXC2064/1–390727645. This research has been supported by the National Science Foundation, Program in the Science of Learning and Augmented Intelligence Award #193683.
